# *Mab_3168c*, a Putative Acetyltransferase, Enhances Adherence, Intracellular Survival and Antimicrobial Resistance of *Mycobacterium abscessus*

**DOI:** 10.1371/journal.pone.0067563

**Published:** 2013-06-28

**Authors:** Sheng-Hui Tsai, Gwan-Han Shen, Chao-Hsiung Lin, Jiue-Ru Liau, Hsin-Chih Lai, Shiau-Ting Hu

**Affiliations:** 1 Institute of Microbiology and Immunology, School of Life Science, National Yang-Ming University, Taipei, Taiwan, R.O.C.; 2 Division of Respiratory and Critical Care Medicine, Department of Internal Medicine, Veterans General Hospital, Taichung, Taiwan, R.O.C.; 3 Institute of Respiratory Therapy, China Medical University, Taichung, Taiwan, R.O.C.; 4 Institute of Nursing Care, Hungkuang University, Taichung, Taiwan, R.O.C.; 5 Department of Life Sciences and Institute of Genome Sciences, School of Life Science, National Yang-Ming University, Taipei, Taiwan, R.O.C.; 6 Center for Molecular and Clinical Immunology, Chang Gung University, Taoyuan, Taiwan, R.O.C.; 7 Department of Medical Biotechnology and Laboratory Sciences, Chang Gung University, Taoyuan, Taiwan, R.O.C.; 8 Research Center of Bacterial Pathogenesis, Chang Gung University, Taoyuan, Taiwan, R.O.C.; Cornell University, United States of America

## Abstract

*Mycobacterium abscessus* is a non-tuberculous mycobacterium. It can cause diseases in both immunosuppressed and immunocompetent patients and is highly resistant to multiple antimicrobial agents. *M. abscessus* displays two different colony morphology types: smooth and rough morphotypes. Cells with a rough morphotype are more virulent. The purpose of this study was to identify genes responsible for *M. abscessus* morphotype switching. With transposon mutagenesis, a mutant with a Tn*5* inserted into the promoter region of the *mab_3168c* gene was found to switch its colonies from a rough to a smooth morphotype. This mutant had a higher sliding motility but a lower ability to form biofilms, aggregate in culture, and survive inside macrophages. Results of bioinformatic analyses suggest that the putative Mab_3168c protein is a member of the GCN5-related N-acetyltransferase superfamily. This prediction was supported by the demonstration that the *mab_3168c* gene conferred *M. abscessus* and *M. smegmatis* cells resistance to amikacin. The multiple roles of *mab_3168c* suggest that it could be a potential target for development of therapeutic regimens to treat diseases caused by *M. abscessus*.

## Introduction

*Mycobacterium abscessus* is a rapid growing mycobacterium. It has emerged as an important pathogen of soft tissue, pulmonary, and disseminated infections in both immunocompromised and immunocompetent patients [1], [2], [3], [4]. The soft tissue infections are mainly due to penetrating trauma or surgery. A study of 86 nontuberculous mycobacterial infections of surgical wound and tympanic membrane in central Taiwan found that 100% of these cases were caused by *M. abscessus* [5], [6], [7], [8].

*M. abscessus* is one of the most drug-resistant, rapid-growing mycobacteria [2], [9], [10]. Like other mycobacteria [11], *M. abscessus* has a complex hydrophobic cell wall that constitutes an efficient permeability barrier. Based on analyses of genomic sequences, *M. abscessus* is predicted to produce β-lactamases, aminoglycoside phosphotransferases, and aminoglycoside acetyltransferases that may confer multiple drug resistance [12]. *M. abscessus* is an intracellular pathogen [13], [14]. In culture, *M. abscessus* exhibits two different colony morphology types referred to as rough and smooth morphotypes [13], [15]. These morphotypes correlate with the virulence of *M. abscessus*, and cells with a rough morphotype are more virulent.

The *mmpL4b* gene in the glycopeptidolipid biosynthesis pathway has been shown to be responsible for switching *M. abscessus* colonies from a smooth to a rough morphotype [16]. In this study, we identified a gene designated *mab_3168c*, whose function was unknown, and found that *mab_3168c* controlled the switching of *M. abscessus* colony morphology from a rough to a smooth morphotype. We also found that *mab_3168c* played a role in biofilm formation, intracellular survival, and resistance to antimicrobial agents.

## Results

### Screening and Identification of *M. abscessus* Mutants with Colony Morphotype Switching

**Figure 1:**
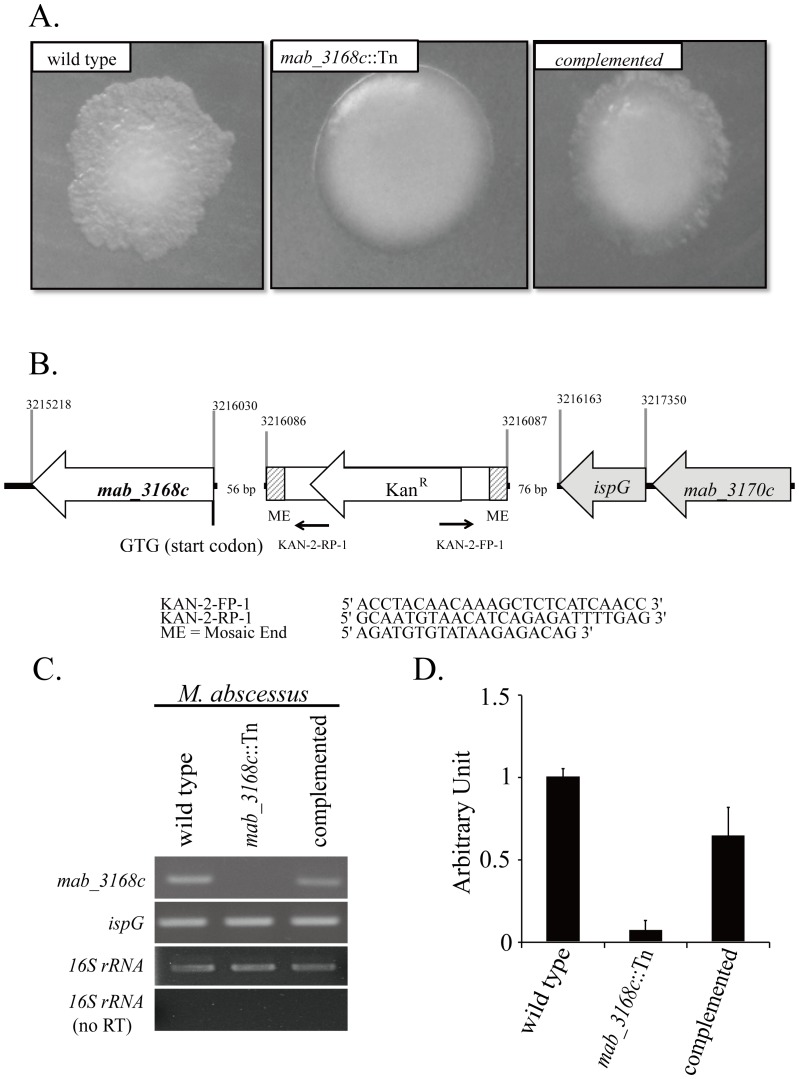
Identification of *mab_3168c*::Tn mutant. (A) Colony morphology of wild type, *mab_3168c*::Tn, and complemented strains of *M. abscessus*. Five days old colonies of *M. abscessus* on 7H11 agar were viewed from the top of the colonies, demonstrating rough and smooth morphologies. (B) Schematic representation of the *mab_3168c* locus and the inserted EZ-Tn*5*™ <Kan^R^> transposon (EPICENTRE®). Numbers shown on top of the figure are nucleotide positions of the *M. abscessus* genome. Primer KAN-2-FP-1 was used to sequence the insertion junctions of Tn*5*. (C) mRNA levels of *mab_3168c* and *ispG* of wild type, *mab_3168c*::Tn mutant, and complemented strains. The 16S rRNA levels were used as the internal control. The 16S rRNA levels determined without reverse transcription served as the negative control for contamination from chromosomal DNA. This experiment was repeated two times. (D) The intensities of the *mab_3168c* mRNA and the 16S rRNA bands were determined by densitometry. The relative intensity value of each sample was obtained by dividing the intensity value of its *mab_3168c* mRNA band by that of its 16S rRNA band. The arbitrary unit of the *mab_3168c* mRNA band of each strain was calculated as the relative density value of its *mab_3168c* mRNA band divided by that of the wild type.

In order to identify the genes involved in *M. abscessus* colony morphotype switching, Tn*5* transposon mutagenesis was performed. A mutant designated *mab_3168c*::Tn (Fig. 1A) that switched its colonies from a rough to a smooth morphotype was identified. Characterization of the genome of this mutant revealed that the transposon was inserted into a place 56 bp upstream from the initiation codon (GTG) of *mab_3168c* (GenBank accession no. NC_010397) and 76 bp downstream from the stop codon of *ispG* (*mab_3169c*) (Fig. 1B). To confirm that this morphotype switching was due to the defect in *mab_3168c,* the intact *mab_3168c* gene was cloned into the *E. coli/mycobacterium* shuttle vector pYUB412A to generate pYUB412A-*mab_3168c* and then introduced into the *mab_3168c*::Tn mutant. This complementation was found to almost completely convert the colonies of the *mab_3168c*::Tn mutant from a smooth back to a rough morphotype (Fig. 1A), suggesting that the *mab_3168c* gene conferred *M. abscessus* the ability to form rough colonies. Since this complementation was not complete (Fig. 1A), RT-PCR was performed to determine mRNA levels of *mab_3168c*. No *mab_3168C* mRNA band was detected in the samples from the *mab_3168c*::Tn mutant (Fig. 1C), and *mab_3168c* mRNA levels in the *mab_3168c* complemented mutant were approximately 60% that of the wild type (Fig. 1D). This result suggested that the incomplete complementation of morphotype was due to sub-optimal expression of the *mab_3168c* gene introduced into the mutant.

As the Tn*5* was inserted 76 bp downstream of the *ispG* gene, *ispG* mRNA levels were also determined and found to be the same in the wild type, *mab_3168c*::Tn mutant, and the complemented strains (Fig. 1C). These results indicated that the Tn*5* insertion inactivated *mab_3168C* but did not affect the expression of its neighboring gene, *ispG* which encodes 4-hydroxy-3-methylbut-2-en-1-yl diphosphate synthase [12], [17]. For simplicity, the *mab_3168c*::Tn mutant and *mab_3168c-*complemented *mab_3168c*::Tn mutant will be referred to as the mutant and the complemented mutant, respectively, hereafter.

### Increased Sliding Motility of the *mab_3168c* Mutant

**Figure 2:**
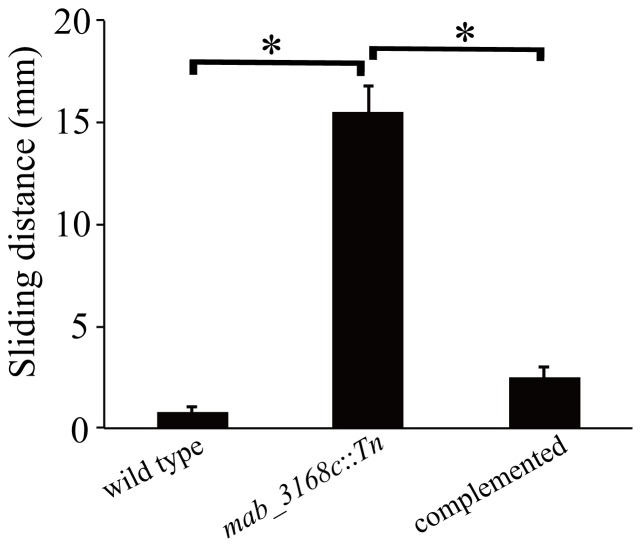
Sliding motility of *M. abscessus* cells. A single colony was inoculated in the center of a plate containing 7H9 medium in 0.3% agar and incubated at 37°C for 5 days. Sliding distances of the 3 strains plotted are means ± standard deviations of three independent colonies. This experiment was repeated three times. Data were analyzed by one-way ANOVA and Fisher’s protected least significant difference (PLSD) test. The asterisk sign (*) represents p<0.05 of *mab_3168c*::Tn versus wild type or *mab_3168c*::Tn versus complemented strain.

Previous studies [18] have shown that the smooth strains of *M. smegmatis* and *M. avium* have a higher sliding ability. Therefore, the motility of the mutant cells on agar plates was examined. As shown in Fig. 2, the wild type *M. abscessuss* cells were non-motile (0.07±0.02 mm), whereas the mutant cells were highly motile (1.55±0.13 mm). The complemented mutant cells regained the non-motile phenotype (0.25±0.05 mm). These results indicated that *mab_3168c* played a major role in inhibiting the motility of *M. abscessus*.

### Decreased Cell Surface Hydrophobicity, Biofilm Formation and Lysozyme Susceptibility of the *mab_3168c* Mutant

**Figure 3:**
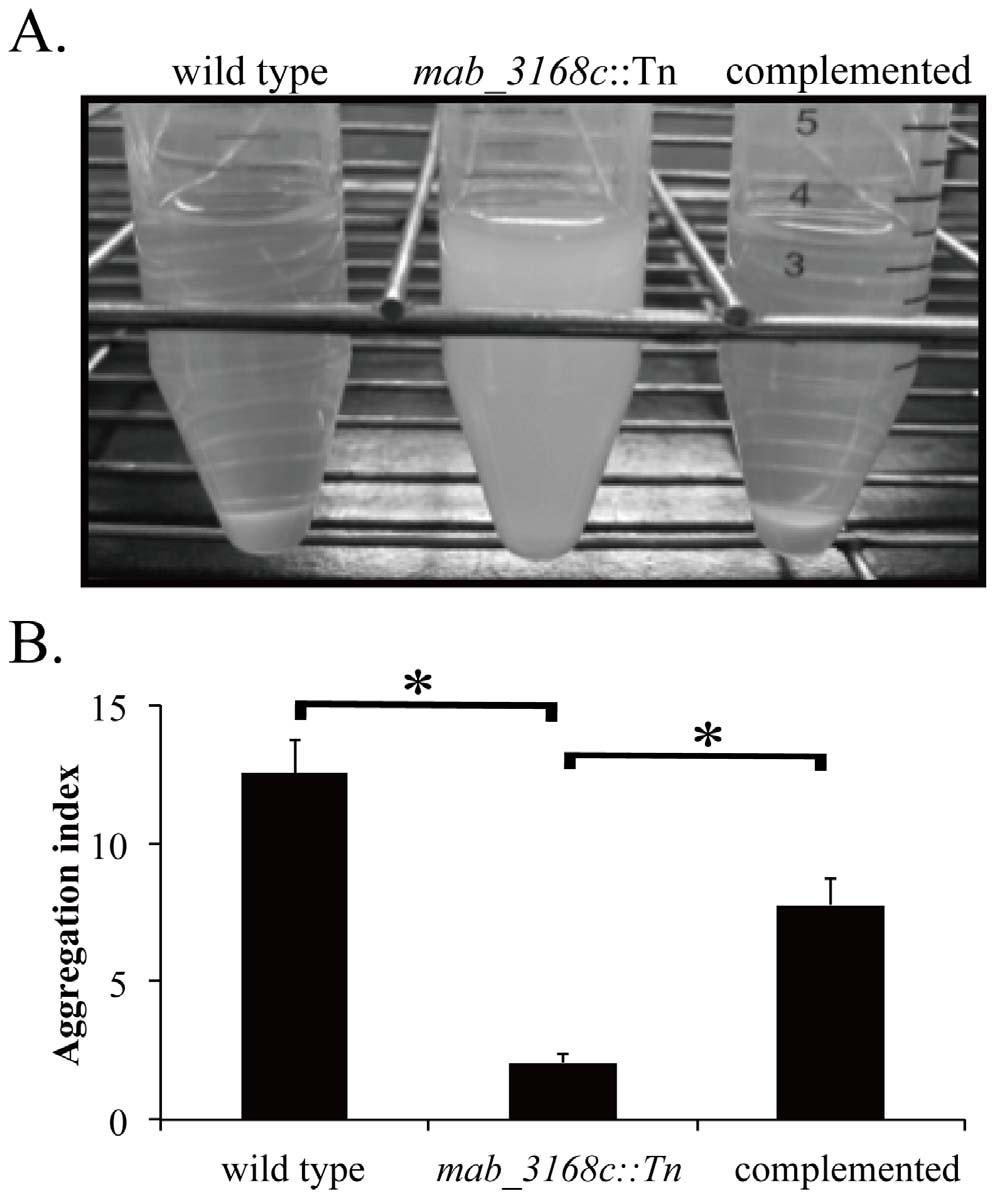
Aggregation of *M. abscessus* cells. (A) *M. abscessus* cells of the 3 different strains were inoculated in 7H9 broth plus 10% OADC at an OD_600_ of approximately 0.1. The cultures were incubated at 37°C for 3 days and then placed at room temperature for 3 hours. (B) Aggregation index of each strain was calculated as the ratio of optical density (OD_600_) of aggregated cells to that of dispersed cells. This experiment was repeated three times. Data were analyzed by one-way ANOVA and Fisher’s PLSD test. The asterisk sign (*) represents p<0.05 of *mab_3168c*::Tn versus wild type or *mab_3168c*::Tn versus complemented strain.

Since the hydrophobicity of cell surface is associated with the sliding activity of mycobacteria [19], experiments were performed to investigate whether the hydrophobicity of the mutant was altered. *M. abscessus* cells of the wild type, mutant, and complemented mutant were grown in liquid Middlebrook 7H9 medium without Tween 80 for 3 days. The mutant exhibited a homogenously dispersed culture, whereas the cultures of both the wild type and complemented mutant had a clear supernatant with cells aggregated in the bottom of the culture tube (Fig. 3A). To adjust for possible variations in growth rates of the 3 strains, an aggregation index of each culture, which is the value of the number of aggregated cells divided by that of dispersed cells, was calculated. The mutant culture was found to have an aggregation index less than 2.5, but the wild type and complemented mutant cultures had aggregation indices of 12±1.2 and 7±0.9, respectively (Fig. 3B). These results indicated that the mutant had a greatly reduced ability to aggregate.

**Figure 4:**
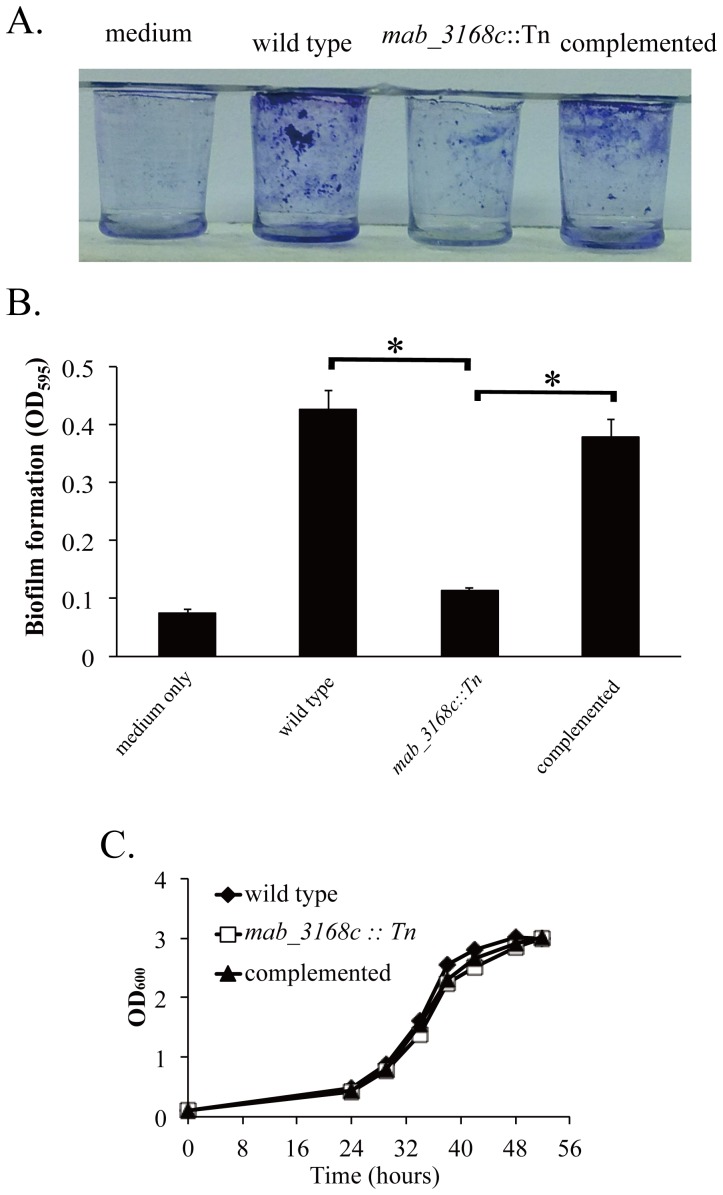
Biofilm-forming capability of *M. abscessus* cells. (A) Cultures of wild type, *mab_3168c*::Tn, and complemented strains were grown on 96-well PVC plates for 6 days. The plates were washed and then stained with crystal violet. (B) Optical density readings of the three strains shown are means ± standard deviations of three independent experiments. Data were analyzed by one-way ANOVA and Fisher’s PLSD test. The asterisk sign (*) represents p<0.05 of *mab_3168c*::Tn versus wild type or *mab_3168c*::Tn versus complemented strain. (C) Growth rates of *M. abscessus* variants. Cells were grown in 7H9 medium supplemented with OADC (10%) and Tween 80 (0.05%), and the OD600 value of each culture was measured at the indicated time points.

As cell surface hydrophobicity is a determinant of adhesion [20], [21], the ability of the mutant to form biofilms was examined. Cells were grown in wells of a 96-well polyvinylchloride plate for 6 days. As shown in Fig. 4A and 4B, substantial formation of biofilms was observed in the wells of wild type *M. abscessus* cultures (OD_595_ = 0.430±0.03). In contrast, biofilm formation by the mutant was diminished (OD_595_ = 0.113±0.005), and the complemented mutant regained the biofilm-forming ability (OD_595_ = 0.378±0.03). These results indicated that the *mab_3168c* gene conferred *M. abscessus* cells the ability to form biofilms by increasing the cell surface hydrophobicity. The reduced ability of the mutant to form biofilm was not due to decreased growth rate because the mutant cells grew equally well as the wild type cells in Middlebrook 7H9 medium (Fig. 4C).

**Figure 5:**
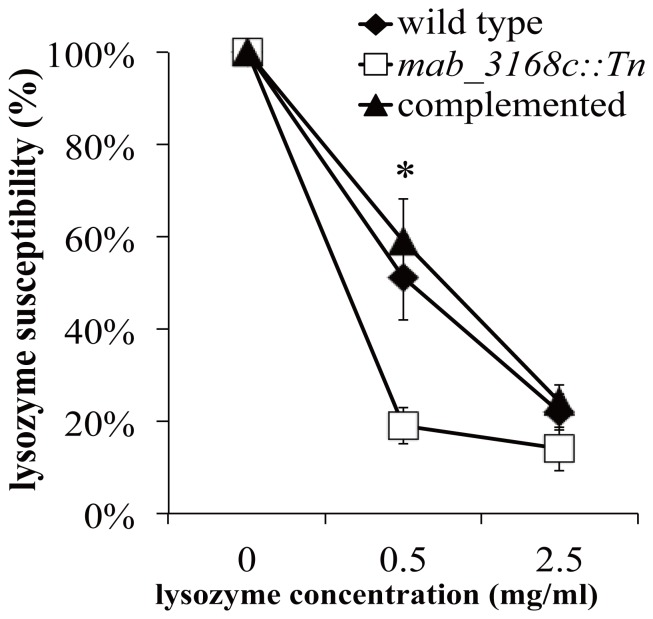
Susceptibility to lysozyme of *M. abscessus* cells. Bacteria were inoculated at 10^7^ CFU/ml into 7H9 broth containing indicated concentrations of lysozyme and incubated at 37°C for 24 hours, followed by enumeration of CFU on 7H11 plate. Results shown are means ± standard deviations of three independent experiments. Data were analyzed by one-way ANOVA and Fisher’s PLSD test. The asterisk sign (*) represents p<0.05 of *mab_3168c*::Tn versus wild type or *mab_3168c*::Tn versus complemented strain.

The susceptibility of the mutant to lysozyme was then assessed. Cells were grown in 7H9 broth containing varying amounts (0, 0.5, and 2.5 mg/ml) of lysozyme. The results showed that the mutant cells were much more susceptible to 0.5 mg/ml of lysozyme than the wild type cells (19% vs. 51% survival) (Fig. 5). Cells of the complemented mutant were found to be almost as resistant to lysozyme as those of the wild type (59% vs. 51% at 0.5 mg/ml lysozyme) (Fig. 5).

### Decreased Intracellular Survival of the *mab_3168c* Mutant in Macrophages

**Figure 6:**
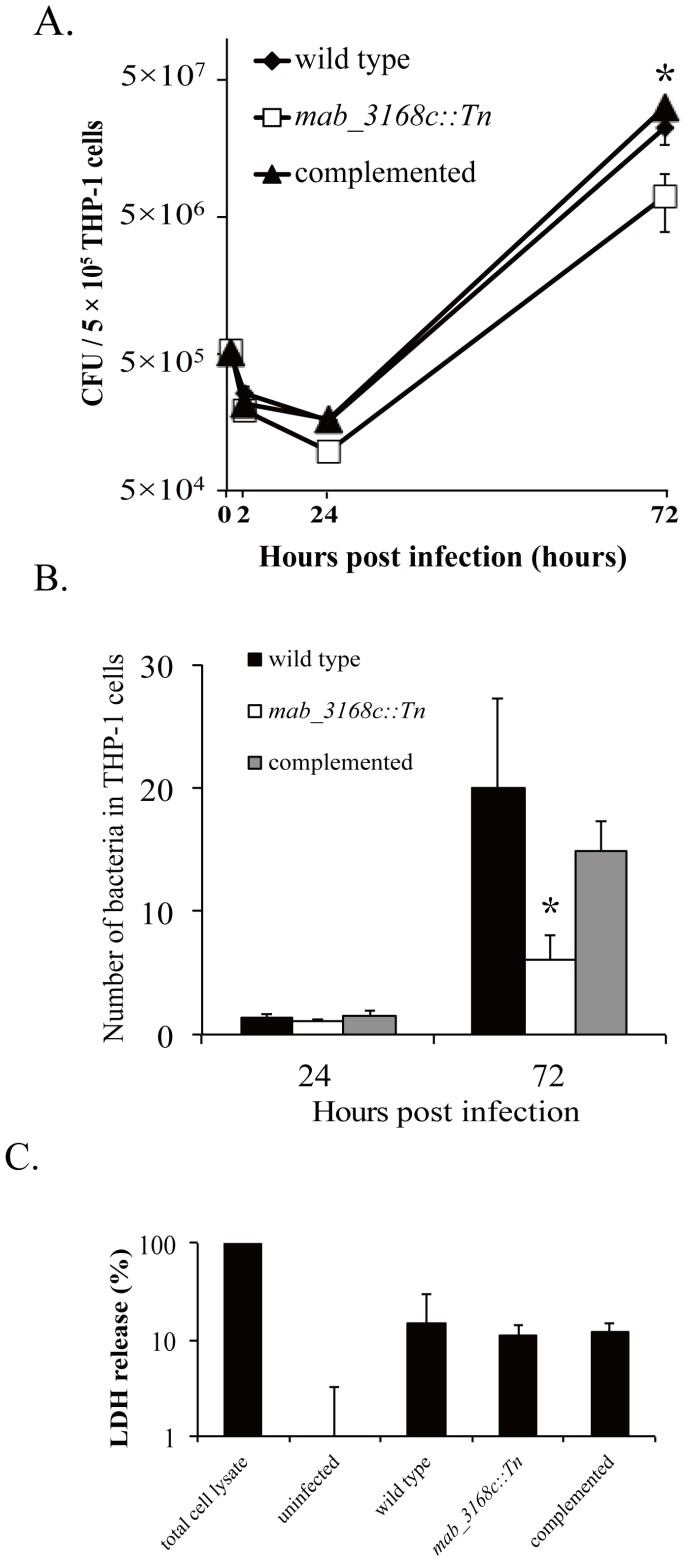
Intracellular survival of *M. abscessus* cells. (A) THP-1 macrophages were co-cultured with wild type, *mab_3168c*::Tn, or complemented *M. abscessus* cells at an MOI of 1 for 2 hours at 37°C. Mycobacterial CFUs in THP-1 cell lysates were then determined at the indicated time points. (B) The number of intracellular *M. abscessus* variants was determined by confocal microscopy. At the indicated time, the *M. abscessus*-infected THP-1 macrophages were fixed and permeabilized prior to staining the intracellular mycobacteria with auramine-O. Organisms in at least 100 cells per slide were counted. (C) Viability of *M. abscessus*-infected THP-1 macrophages. THP-1 macrophages were infected with wild type, *mab_3168c*::Tn mutant, and complemented mutant for 72 hours. The levels of LDH activity in the culture supernatants were measured. Results shown are means ± standard deviations of three independent experiments. Data were analyzed by one-way ANOVA and Fisher’s PLSD test. The asterisk sign (*) represents p<0.05 of *mab_3168c*::Tn versus wild type or *mab_3168c*::Tn versus complemented strain.

The correlation between lysozyme susceptibility and intracellular survival of *M. abscessus* cells was then evaluated. THP-1 macrophages were infected with wild type, mutant, and complemented mutant at a multiplicity of infection (MOI) of 1. At 2, 24, and 72 hours post infection, the number of *M. abscessus* cells survived inside the macrophages was determined by CFU counts. No significant difference in macrophage intracellular survival was observed at 2 and 24 hours after infection. However, the mutant was found to survive more poorly at 72 hours post infection with a lower CFU count [(7.0±3.3)×10^6^] than the wild type [(2.22±0.56)×10^7^] and the complemented mutant [(3.2±0.13)×10^7^] (Fig. 6A). To confirm this result, confocal microscopy was performed to enumerate intracellular mycobacteria. The result showed that the number of *mab_3168c::Tn* mutant was significantly lower (average 6 vs. 20 organisms per cell) than that of wild type and complemented (approximately 16 organisms per cell) strains at 72 hours post infection (Fig. 6B). To show that this lower intracellular organism count was not due to death of infected THP-1 cells, the viability of uninfected and infected cells was assessed by determining LDH levels in culture supernatants. As shown in Fig. 6C, similar levels of LDH were observed in the culture supernatants of THP-1 cells infected with wild type, mutant, and complemented mutant (approximately 10% of that of total cell lysate). Very little LDH was detected in the culture supernatant of uninfected cells. Taken together, these results demonstrated that *mab_3168c* was required for intracellular survival of *M. abscessus* in macrophages.

### Bioinformatics Analysis of *mab_3168c*

Bioinformatic analyses of the Mab_3168c protein were performed to investigate its possible functions (data not shown). A large number of proteins with a significant homology to the 270-aa Mab_3168c protein were found by BLASTP. Most of these proteins were members of the GCN5-like N-acetyltransferase (GNAT) superfamily. Reversed Position Specific Blast (RPS-BLAST) analyses revealed the presence of an acetyltransferase domain of the pfam00583 family. This domain extends from residues 205 to 259 of the putative Mab_3168c protein and contains the consensus sequence V/I-x-x-x-x-Q/R-x-x-G-x-G/A of acetyltransferases [22]. Results of 3D-PSSM prediction also showed that Mab_3168c bears a strong structural similarity to several acetyltransferases of the GNAT family, especially to the aminoglycoside 6′-N-acetyltransferase of *Enterococcus faecium*. These results suggest that Mab_3168c may function as an acetyltransferase.

### Increased Susceptibility of the Mutant to Amikacin

**Figure 7:**
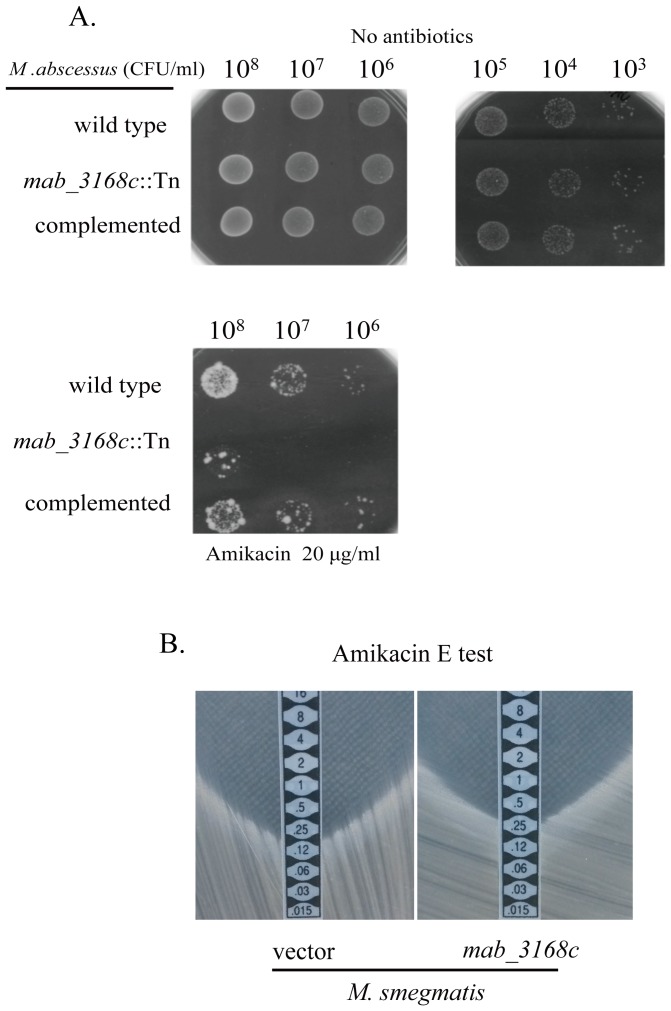
Contribution of *mab_3168c* to aminoglycoside resistance. (A) Anti-mycobacterial effects of amikacin against various *M. abscessus* strains were determined by plating indicated number of cells on 7H11 agar plates. The experiments were repeated 3 times. One representative picture of each of the 3 days old cultures without antibiotics and the 5 days old cultures with antibiotics is shown. (B) Amikacin susceptibility of *M. smegmatis* containing pYUB412A (vector) or pYUB412A-*mab_3168c* (*mab_3168c*) was examined by the E test. The experiment was repeated 3 times. One representative picture of the E test of each of the 5 days old cultures is shown.

As the aminoglycoside 6′-N-acetyltransferase of *Enterococcus faecium* contributes to its aminoglycoside resistance [23], the possibility that *mab_3168c* conferred *M. abscessus* antibiotic resistance was examined. Cells of the wild type, mutant, and complemented mutant were grown on 7H11 agar plates with or without rifampin, ciprofloxacin, or amikacin. Other aminoglycosides such as kanamycin, neomycin, paromomycin, ribostamycin, and gentamycin B were not tested because the transposon used for mutagenesis contains the kanamycin-resistance gene, which also confers resistance to these aminoglycosides. No difference in susceptibility to rifampin and ciprofloxacin was observed among the three different strains (data not shown). However, cells of the mutant were more sensitive to amikacin than those of the wild type and the complemented mutant (Fig. 7A). In an overnight culture inoculated with 10^7^ or 10^6^ CFU/ml, the growth of mutant cells was completely inhibited by 20 µg/ml of amikacin, and the growth of the culture inoculated with 10^8^ was inhibited by 6.1±4.4% with a survival rate of (4.1±3.0)×10^−5^, which was calculated as the CFUs on the amikacin plate divided by those on the plate without amikacin. Cells of the complemented mutant were almost as resistant as those of the wild type to 20 µg/ml of amikacin with a survival rate of (6.7±0.5)×10^−4^ and (7.8±2.0)×10^−4^, respectively (Fig. 7A). To confirm amikacin susceptibility, the E test was carried out. As shown in Table 1, the amikacin MICs of both the wild type and complemented strains were 4 µg/ml, but the MIC of the *mab_3168c::Tn* mutant was only 2 µg/ml.

**Table 1:** Amikacin E-test.

Strain	MIC (µg/ml)
*M. abscessus*	
wild type	4
* mab_3168c::Tn*	2
* Complemented*	4
*M. smegmatis*	
vector only	0.12
* mab_3168c*	0.25

To further confirm that the *mab_3168c* gene conferred resistance to amikacin, it was introduced into a different mycobacterium, *M. smegmatis,* and then assayed the transformants for their susceptibility to amikacin. The MIC value of *M. smegmatis* cells containing pYUB412A-*mab_3168c* was two-fold higher than those containing the vector pYUB412A (0.25 vs. 0.12 µg/ml) (Fig. 7B and Table 1).

## Discussion

In this study, we showed that loss of *mab_3168c* expression resulted in alterations in colony morphology, cell surface hydrophobicity, sliding motility, biofilm forming ability, amikacin and lysozyme resistance, and intracellular survivability of *M. abscessus*. Although bioinformatic analyses revealed the presence of a sequence motif characteristic of the GCN5-related N-acetyltransferase (GNAT), it remains to be determined whether the putative Mab_3168c protein is an acetyltransferase as our repeated attempts to express the *mab-3168c* gene have not been successful.

The relationship between an acetyltransferase and colony morphology was first determined in *M. smegmatis* [24]. In this organism, disruption of the *atf1* gene was found to cause its colonies to switch from a smooth to a rough morphotype [24]. The *atf1* gene encodes an *O*-acetyltransferase which is believed to acetylate glycopeptidolipids (GPLs) [24]. However, we did not observe any differences in the lipid profiles of both the wild type and *mab_3168c* mutant cells by thin-layer chromatography (TLC) and MALDI-TOF analysis (data not shown).

It was unexpected to observe that the cell wall lipid profiles of the wild type with a rough morphotype and the mutant with a smooth morphotype had the same lipid profiles as GPL is believed to make mycobacterial colonies smooth [16], [19], [24], [25]. One possibility is that other cell wall components also affect colony morphology. In mycobacteria, UDP-N-acetylglucosamine (UDP-GlcNAc), a major component of peptidoglycan, is synthesized by the GlmU protein, which is a glucosamine-1 phosphate acetyltransferase. Mutation of *glmU* has been shown to impair the synthesis of peptidoglycan and reduce the growth of *M. smegmatis* [26]. It is possible that instead of affecting GPL production, Mab_3168c affects the synthesis of other cell wall components, similar to the GlmU protein.

Another gene that has been shown to be associated with colony morphotype of *M. abscessus* is *mmpL4b*, which encodes a membrane protein [16]. Deletion of this gene renders *M. abscessus* unable to produce GPL and to form smooth colonies. The Δ*mmpL4b* mutant also loses the sliding motility and the ability to form biofilms. However, it survives better in macrophages. In this study, we found that the *mab_3168c* mutant formed smooth colonies, gained the sliding motility, but lost the ability to survive inside macrophages. These three properties are opposite to those of the Δ*mmpL4b* mutant. However, similar to the Δ*mmpL4b* mutant, the *mab_3168c* mutant also lost the ability to form biofilms. These results suggest that biofilm formation is controlled by multiple mechanisms, and both *mmpL4b* and *mab_3168c* genes regulate biofilm formation. Further support of this hypothesis is the finding that inactivation of the *lsr2* gene renders *M. smegmatis* hyper motile and unable to form biofilms [27], very similar to the *mab_3168c* mutant. The *lsr2* mutant also has no changes in cell wall lipid profiles [27], [28]. In *M. tuberculosis*, Lsr2 is a nucleoid-associated protein, similar to the histone-like nucleoid structural protein H-NS [29], [30] and is involved in the regulation of cell wall synthesis [31] as well as transcription suppression of many genes [30]. Although *lsr2* and *mab_3168c* mutants are phenotypically similar, *lsr2* and *mab_3168c* genes are distinct with no significant sequence homologies.

The *mab_3168c* gene was shown to confer *M. abscessus* resistance to amikacin which is a semisynthetic aminoglycoside derived from kanamycin. Many bacteria that are resistant to gentamicin and tobramycin are sensitive to amikacin. Therefore, amikacin is often used to treat *M. abscessus* infections [32]. Aminoglycoside resistance may be due to decreased cell permeability, alterations in ribosome binding, or inactivation by aminoglycoside modifying enzymes [33]. It is likely that *mab_3168c* is involved in cell wall synthesis making *M. abscessus* cells less permeable to amikacin. It is also possible that Mab_3168c acetylates amikacin rendering it inactive. In addition to Mab_3168c, *M. abscessus* may also produce other enzymes that can inactivate antibiotics as analyses of genomic sequences revealed its potential to produce β-lactamases and aminoglycoside converting enzymes including aminoglycoside O-phosphotransferases and aminoglycoside N-acetyltransferases [12], [34], [35]. This property could explain the multiple drug resistance of *M. abscessus*.

We also found that inactivation of *mab_3168c* decreased the ability of *M. abscessus* to survive inside macrophages. This defect is likely due to increased susceptibility to lysozyme. This possibility is supported by the finding that disruption of the aminoglycoside 2′-N-acetyltransferase gene, *acc(2′)-Id*, renders *M. smegmatis* susceptible to lysozyme [36].

In conclusion, compared to the known characteristics of different members of the GNAT superfamily, we predict that Mab_3168c is an N-acetyltransferase. Inactivation of *mab_3168c* may cause changes in the structure of the cell wall, resulting in a pleiotropic phenotype of *M. abscessus* with altered colony morphotype, increased sliding motility, reduced cellular aggregation and ability to survive inside macrophages, and increased susceptibility to amikacin. Since *mab_3168c* plays a role in many different cellular functions, it could be a good target for development of drugs against *M. abscessus.*

## Materials and Methods

### Bacterial Strains and Culture Condition

*M. abscessus* strain cs1c [5] was obtained from Institute of Respiratory Therapy, China Medical University, Taichung, Taiwan. Mycobacteria were grown at 37°C on Middlebrook 7H11 (Difco, USA) agar supplemented with 10% OADC (Oleic acid-bovine albumin-dextrose-catalase) (Becton Dickinson, USA) or in Middlebrook 7H9 broth (Difco, USA) containing 10% OADC, 0.2% glycerol, and 0.05% Tween 80. Colony morphology was examined at 50X magnification using a Nikon SMZ645 Stereo Microscope. Apramycin and hygromycin were used when required at concentrations of 30 and 50 µg/ml, respectively.

### Transposon Mutagenesis and Genetic Analysis of Mutants

The *M. abscessus* transposon mutant library was generated using the EZ-Tn*5*™ <KAN-2>Tnp Transposome™ Kit (EPICENTRE, USA). Approximately 2000 mutants were screened to detect the ones with altered colony morphology. The Tn*5* insertion site in the chromosome was identified by inverted PCR using KAN-2 FP-1 forward and KAN-2 RP-1 reverse primers (Fig. 1B) and by DNA sequencing.

### Complementation of Mutants with *mab_3168c*

The mycobacterial shuttle vector pYUB412 [37] was used as a backbone in which the hygromycin-resistance gene was replaced by an apramycin-resistance gene to construct pYUB412A. Genomic DNA of *M. abscessus* was used as template for PCR to amplify the region encompassing the whole-length *mab_3168c* gene and 1 kb upstream of the gene, using the primer pair *mab_3168c*-F (5′-AGGTATACCATCTTCGCGGCGAT-3′) and *mab_3168c*-R (5′-AGCTCGAGTTAGCTGACGGGGA-3′), containing BstZ17I and XhoI sites (underlined), respectively. The resulting 1.8-kb DNA fragment was cloned into pYUB412A between ZraI and SalI sites, generating plasmid pYUB412A-*mab_3168c*. For complementation, pYUB412A-*mab_3168c* was introduced into *mab_3168c*::Tn mutant by electroporation. Electrocompetent *M. abscessus* cells were prepared by growing them to mid-log phase. The cells were harvested, washed in 10% glycerol and then resuspended in cold 10% glycerol at a concentration of 10^7^ cells/µl.

### Determination of mRNA Levels by RT-PCR

*M. abscessus* cells were grown to mid-log phase. The cells were harvested, resuspended in 1 ml TRIzol (Invitrogen, USA), and lysed by beating with 0.1 mm silica/zirconium beads in a Mini-beadbeater (Biospec, USA). RNA was isolated by conventional phenol/chloroform extraction and isopropanol precipitation. RT-PCR was performed to examine *mab_3168c* and *ispG* mRNA expression by using the following primer pairs: 16S rRNA, 5′-TCAGCTTGTTGGTGGGGTAATGG-3′ forward and 5′-ACGCGACAAACCACCTACGAGCT-3′ reverse; *mab_3168c*, 5′-ACCGCAGGCGTGGCGGCGAT-3′ forward and 5′-TTAGCTGACGAGGACCGTCG-3′ reverse; *ispG*, 5′-TTTGGAGCGCTGTTGTCCAA-3′ forward and 5′-CCCGCGCTGACGGCGTTGGC-3′ reverse.

### Sliding Motility Test

One colony of each mycobacterium was inoculated in the center of a motility plate, consisting of Middlebrook 7H9 with 0.3% agar. The inoculated plates were incubated at 37°C for 5 days [18]. The sliding distance was measured in millimeters.

### Aggregation Capability Assay

Mycobacterial cells were incubated in a tube containing 5 ml of Middlebrook 7H9 broth at a concentration of OD_600_ = 0.1 and incubated on a shaker at 37°C for 3 days. After allowing the culture tube to stand still for 3 hours, the upper portion of the culture containing dispersed cells was removed, and its OD_600_ value was determined. The OD_600_ of the bottom portion of culture was measured after the aggregated cells had been completely suspended by vortexing with glass beads of 4.5 mm in diameter (Biospec, USA) as described previously [13], [38], [39]. The aggregation index was calculated as the ratio of optical density of aggregated cells to that of dispersed cells.

### Biofilm Formation

Mycobacterial cells at a concentration of OD_600_ = 0.1 were inoculated in 100 µl of Middlebrook 7H9 broth in each well of a sterile 96-well, flat-bottom polyvinylchloride plate (BD, USA). After 6 days of incubation, the medium in each well was removed, and the wells were washed with sterile PBS to remove non-adherent cells. The wells were then stained with 0.5% (wt/vol) crystal violet for 1 hour. After washing with PBS, the stained biofilms were photographed. To quantitate cells, cells in the biofilm were suspended in 100% ethanol, and the OD_595_ value of the cell suspension was determined [40].

### Lipid Extraction and Analysis

Total lipids from mycobacterial cells of plate-grown cultures were extracted with chloroform/methanol (2∶1, v/v) at 56°C for 60 min with sonication. The extracted lipids were spotted on an aluminum-backed silica gel_60_ TLC plate (MERCK, German) and resolved with a solvent containing chloroform and methanol at a ratio of 90∶10 (v/v) or chloroform, methanol, and water at a ratio of 100∶16:2 (v/v) or 60∶16:2 (v/v) as previously described [41], [42]. To visualize lipids, the plate was sprayed with 1% 1-naphthol, 5% H_2_SO_4_ in ethanol and then charred with a heat gun until spots with hues characteristic of different lipid classes appeared. The mass of each lipid species was determined by matrix-assisted laser desorption ionization-time-of-flight (MALDI-TOF) spectrometry with a pulse laser emitting at 337 nm. Samples were mixed with 2,5-dihydroxybenzoic acid as the matrix and analyzed in reflectron mode with an accelerating voltage of 25 kV.

### Lysozyme Susceptibility Assay

*M. abscessus* cells (10^7^/ml) were inoculated into 7H9 broth containing various concentrations of lysozyme (0.5 mg/ml and 2.5 mg/ml) and incubated at 37°C for 24 hours, followed by enumeration of CFU on 7H11 agar plates.

### Infection of Human Macrophages

THP-1 cells, a human acute monocytic leukemia cell line, were obtained from the American Type Culture Collection (ATCC) and cultured in RPMI 1640 medium supplemented with 10% fetal bovine serum (FBS, GIBCO) at 37°C in a humidified CO_2_ incubator. THP-1 cells were differentiated into adherent macrophages by adding 500 ng/ml of phorbol-12-myristate-13-acetate (PMA) to the culture. Two days after addition of PMA, the cells were infected with mycobacteria at a multiplicity of infection (MOI) of 1 for 2 hours at 37°C. The infected macrophages were washed with sterile PBS to remove extracellular mycobacteria, lysed with 1% Triton X-100, and then plated on 7H11 agar plates to determine the colony forming unit of intracellular mycobacteria as describe previously [43], [44], [45].

Viability of *M. abscessus*-infected THP-1 macrophages was evaluated by measuring the levels of lactate dehydrogenase (LDH) in culture supernatants. THP-1 macrophages were infected with wild type, *mab_3168c*::Tn mutant, and complemented mutant. The levels of LDH activity in the culture supernatants were determined using a CytoTox 96 assay kit (Promega, USA) according to manufacturer’s protocol.

### Confocal Microscopy

Infected macrophages were fixed with 4% paraformaldehyde for 15 min and permeabilized with 0.1% Triton-X 100 for 20 min. The intracellular mycobacteria were stained with auramine (Sigma, USA) for 20 min at 25°C, treated with 0.5% acid alcohol for 3 min, and then examined under a confocal laser scanning microscope (Leica SP5 confocal Microscopy equipped with a 100X NA1.4 objective lens).

### Antimicrobial Susceptibility Test

Susceptibility to amikacin was determined by the E test. *M. abscessus* and *M. smegmatis* cells were incubated in 7H9 medium until their culture turbidity reached McFarland standard of 1.0 (∼3×10^8^ CFU/ml). This cell suspension was then spread on a 7H11 agar plate (10 cm) supplemented with 10% OADC using a cotton swab. An amikacin E test strip (Oxoid) was then placed on the plate, and the plate was incubated for 3–5 days until the MIC was read. The value shown on the strip at the place where the strip intersected the growth inhibition zone was the amikacin MIC of the organism tested. The MIC data presented were average of duplicate determinations.
